# A Comparative Study on the Influence of Undersampling and Oversampling Techniques for the Classification of Physical Activities Using an Imbalanced Accelerometer Dataset

**DOI:** 10.3390/healthcare10071255

**Published:** 2022-07-05

**Authors:** Dong-Hwa Jeong, Se-Eun Kim, Woo-Hyeok Choi, Seong-Ho Ahn

**Affiliations:** 1 Department of Artificial Intelligence, The Catholic University of Korea, Bucheon 14662, Korea; hohoan05@gmail.com; 2 School of Computer Science and Information Engineering, The Catholic University of Korea, Bucheon 14662, Korea; seeun4209@icloud.com (S.-E.K.); uoo1325@naver.com (W.-H.C.)

**Keywords:** physical activity, accelerometer, ensemble method, random forest, bootstrap aggregating (bagging), adaptive boosting, undersampling, oversampling

## Abstract

Accelerometer data collected from wearable devices have recently been used to monitor physical activities (PAs) in daily life. While the intensity of PAs can be distinguished with a cut-off approach, it is important to discriminate different behaviors with similar accelerometry patterns to estimate energy expenditure. We aim to overcome the data imbalance problem that negatively affects machine learning-based PA classification by extracting well-defined features and applying undersampling and oversampling methods. We extracted various temporal, spectral, and nonlinear features from wrist-, hip-, and ankle-worn accelerometer data. Then, the influences of undersampilng and oversampling were compared using various ML and DL approaches. Among various ML and DL models, ensemble methods including random forest (RF) and adaptive boosting (AdaBoost) exhibited great performance in differentiating sedentary behavior (driving) and three walking types (walking on level ground, ascending stairs, and descending stairs) even in a cross-subject paradigm. The undersampling approach, which has a low computational cost, exhibited classification results unbiased to the majority class. In addition, we found that RF could automatically select relevant features for PA classification depending on the sensor location by examining the importance of each node in multiple decision trees (DTs). This study proposes that ensemble learning using well-defined feature sets combined with the undersampling approach is robust for imbalanced datasets in PA classification. This approach will be useful for PA classification in the free-living situation, where data imbalance problems between classes are common.

## 1. Introduction

Physical activity (PA), which can be defined as all types of body movements produced by skeletal muscles that require energy expenditure, plays a key role in the well-being of both mental and physical health. Nowadays, due to the increased manifestation of a sedentary lifestyle, many people are facing elevated health risks [1]. Both moderate PAs, which include daily activities such as walking, and vigorous PAs, such as exercises and sports, can improve human health and quality of life. Furthermore, many studies have reported that PA can mitigate the risks of metabolic disorders [2], cardiovascular diseases [3], cancer [4], neurological diseases [5], and psychiatric disorders [6,7]. In this regard, it is important to monitor PA in daily life to provide an appropriate intervention to encourage exercise. Recently, wearable devices, or wearables, have been widely used with the advancement of smart devices and embedded sensors. The most commonly used wearables are wrist-worn smartwatches which can collect the user’s physiological data while performing the basic functions of smartphones [8,9,10]. Wearable sensors can also be attached to various body positions of the torso (chest, waist, and hip) [11,12], lower limbs (legs and feet) [13], upper limbs (forearm and finger) [14], or head (scalp and ear) [15,16,17], depending on their purpose.

Accelerometers, which measure acceleration in three dimensions—vertical, mediolateral, and anterior-posterior—have often been used in wearable sensors to measure PA objectively in the free-living environment. Numerous studies have attempted to distinguish PA from sedentary behaviors by adopting a cut-point approach with the accelerometry data [18,19,20,21,22]. Cut-points are generated to differentiate moderate-to-vigorous physical activity (MVPA) by finding the optimal accelerometer activity counts that best correspond to the energy expenditure [23,24,25]. However, because the cut-point approach has limitations in differentiating the activities of daily living from similar patterns of acceleration, it can lead to a biased estimation of energy expenditure [26,27]. For example, the energy expenditure of climbing stairs is nearly twice that of walking on a flat surface, although both activities produce similar patterns of acceleration [28]. Therefore, it is more important to recognize the specific type of performed activity rather than its duration or intensity to quantify PA. Since the classification of a PA type enables more precise estimation of energy expenditure and provides more informative human behavioral data, wearable PA recognition can be applied to various fields, including healthcare and human–machine interfaces [29,30,31].

In recent decades, various machine learning (ML) methods including deep learning (DL) techniques, which can learn the patterns of linear or nonlinear features extracted from the raw accelerometer data, have been adopted for the classification of human behavior. Most studies have utilized various ML and DL methods to classify six different types of PA, including walking on level ground, walking upstairs, walking downstairs, sitting, standing, and lying, using an open database presented by the UCI machine learning repository [32,33,34]. In the subject-specific paradigm, where an independent classifier is trained and evaluated within each subject, ML classifiers such as k-nearest neighbors (k-NN), multilayer perceptron (MLP), and random forest exhibited great performance [35,36,37]. In regard to the PA classification, the performance of the subject-specific paradigm was better than that of the group-level paradigm, which used the model pretrained with data from other subjects, due to the different sensor locations and behavioral patterns of each subject [38,39,40]. In the group-level paradigms, DL approaches including convolutional neural networks (CNN) resulted in high PA classification accuracy [41,42,43]. For example, Ronao and Cho achieved 94.79% performance for the classification of six PAs using a CNN in the UCI dataset [41]. Ignatov also reported that the CNN outperformed RF and k-NN in the UCI and WISDM datasets [42]. The author also demonstrated that the CNN approach led to successful classification in cross-dataset evaluation. Hassan et al. proposed a deep belief network (DBN)-based PA classifier that automatically extracts features from raw sensor data to classify 12 different exercises with 97.5% accuracy [43]. Although these studies demonstrate the enhanced performance of DL-based classifiers compared with that of standard ML-based models, some studies reported that the ML approach with a well-defined feature set could outperform DL approaches when the resource, such as a hardware specification or the amount of the dataset, is limited. For instance, Montoye et al. proposed that RF outperformed other ML models, including neural networks, in classifying 21 PAs [44].

Although many studies demonstrated that the ML and DL techniques were useful for predicting a wide range of daily living activities, most studies have utilized the accelerometer data collected in the laboratory environment where participants were instructed to perform certain activities. Therefore, in these controlled settings, the length of the data for each PA could be balanced. However, in a free-living situation, people spend most of their time indulging in a few activities such as sedentary or light behavior, including walking on level ground. On the other hand, people relatively do not engage much in moderate or vigorous PAs, including ascending stairs or running. Since the ML and DL approaches require a large amount of data for each class, the data imbalance between each PA could pose a challenge in building robust ML classifiers. In general, when using an imbalanced dataset, ML models are prone to making biased predictions toward the majority class. In many studies on PA classification, the recognition rate of the minority class was much lower than that of the majority class despite high overall accuracy [38,39,45,46].

In this regard, this study aims to comprehensively examine the influence of the data imbalance between PA classes by adopting multiple ML and DL methods after performing data balancing techniques. Undersampling and oversampling methods are often used in ML and DL studies to adjust the class distribution. Undersampling methods reduce the size of the majority class by randomly discarding samples in the majority class. The oversampling method is more frequently adopted in ML and DL studies. Random oversampling, in which random samples in the minority class are simply duplicated, can induce an overfitting problem. Therefore, the synthetic oversampling techniques, which generate novel samples that have similar statistical properties to the samples in the minority class, are generally more preferred.

This study utilized an open database presented in PhysioNet [47], which contains labeled raw accelerometer data during walking on level ground, ascending and descending stairs, and driving. Since there was a huge variation between the durations of each PA (walking on level ground: 262.80 min (26.88%), ascending stairs: 46.65 min (4.67%), descending stairs: 44.59 min (4.56%), and driving: 623.58 min (63.79%)), this dataset was considered adequate to examine the influence of the data imbalance problem. In the original study that provided this dataset, the decision trees (DTs) were trained with spectral features to identify different types of walking [39]. Despite the high performance in the subject-specific paradigm, relatively poor performance was shown in the group-level paradigm due to the poor recognition rate of the minority class. While the classification of walking and sedentary behaviors such as driving exhibited relatively high performance, that of different walking types was more challenging due to similar PA patterns [22,39]. In other studies, the recognition rate of walking upstairs and walking downstairs was much lower than that of other PAs even with the balanced distribution of PAs [41,45,46].

We aim to investigate the influence of multiple classification techniques fused with data sampling techniques on the imbalanced PA dataset. We propose a feature set consisting of temporal, spectral, and nonlinear features, which have been widely used not only in PA classification but also in the analysis of time series data such as biosignals. We also propose that an ensemble learning method that makes predictions with multiple decision trees (DTs) efficiently captures multi-domain features related to the PA classification and is robust to small datasets. The main contribution of this study can be described as follows:

1. Undersampling and oversampling with ensemble methods could resolve the class imbalance between different PA types;
2. When using ensemble methods with well-defined features, undersampling was more efficient than the oversampling approach, since it requires a low computational cost while maintaining high classification performance;
3. Ensemble methods based on decision trees (DTs) successfully made decision criteria based on the multi-domain features and the locations of the accelerometers.

The remainder of this paper is organized as follows. Section 2 contains the data description and proposed methods, including feature extraction, data sampling, ensemble learning, and comparative ML models. Section 3 describes the classification performance based on data sampling techniques and ML models, along with the contribution of each feature set to the PA classification. Finally, in Section 4 and Section 5, the main findings, implications of this study, and the conclusion are described.

## 2. Materials and Methods

### 2.1. Data Description

For this study, the open database of raw accelerometry data of 32 healthy subjects (13 males and 19 females) was adopted from PhysioNet [46]. In the database, three-axial acceleration was measured from the left wrist, left hip, left ankle, and right ankle. All subjects but one, who identified as ambidextrous, were right-handed. Their average age, height, and weight were 39.03 ± 8.84 years (23–54), 68.31 ± 4.30 inches (58–76), and 169.69 ± 49.61 lbs (100–310), respectively. Throughout the experiment conducted at Indiana University, all subjects wore ActiGraph GT3X+ accelerometer devices (Actigraph, Pensacola, FL, USA) for data collection while performing various PAs. The four devices were attached on the outside of both ankles, the top side of the left wrist similar to a regular watch, and the belt of the subject on the left hip. The sampling rate was 100 Hz for all devices, and all devices were synchronized. Desynchronization between the devices existed, but no serious desynchronization was observed.

The experiment started with a walking session on a trail of 0.66 miles, where the subjects were asked to walk on level ground, descend stairs, and ascend stairs for a total of 9–13.5 min. Walking on level ground was repeated five times, while descending and ascending stairs were repeated six times. In the following driving session, the subjects drove on a city road and highway trails of 12.8 miles for approximately 18–30 min. Throughout the experiment, the subjects were instructed to walk at their usual pace and to drive along a predetermined route to simulate free-living activities. To identify the exact time points, the subjects were told to clap three times at the start and end of each activity, which induced a corresponding amplitude change in the accelerometry data. The periods of clapping and non-study activity, which refers to a few seconds before and after each activity, were excluded from this study because they were only used to mark the time points of each trial.

**Figure 1:**
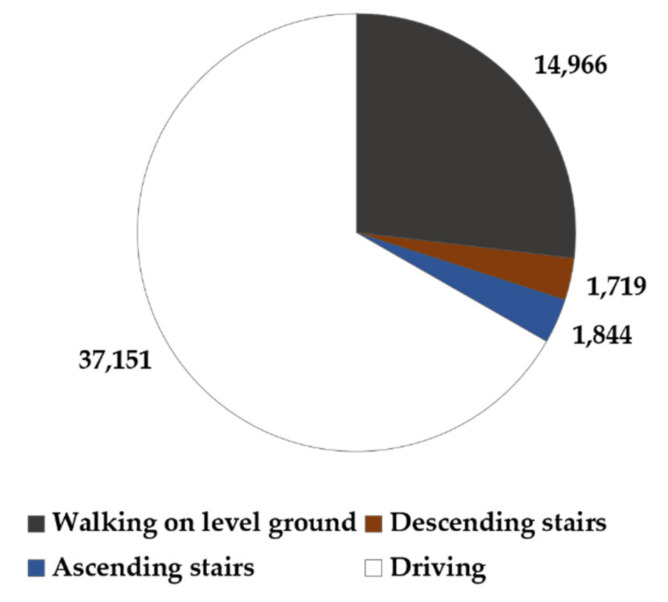
A total number of 5-s epochs in the PA dataset after excluding epochs of transition where two different PAs overlap.

Classification results were provided every 5 s because the features were extracted from a 5-s window. Epochs of transition where two different PAs overlapped were excluded from the analysis. As a result, a total of 55,680 epochs for 4 PAs were collected from 32 subjects. The ratio of each PA was 66.72% for driving, 26.88% for walking on level ground, 3.31% for ascending stairs, and 3.09% for descending stairs. The data distribution for each PA class is shown in Figure 1.

In this study, a group-level paradigm was adopted to classify four PAs, including walking on level ground, ascending stairs, descending stairs, and driving. The accelerometer data recorded from three different body parts were independently analyzed to see if the place of sensor attachment influenced the PA classification. Accelerometer data were randomly divided into training and test data at a 7:1 ratio. Thus, among 32 subjects, 28 were assigned to the training set, while the remaining subjects were assigned to the test set. This process was repeated eight times by permuting the training and test data set. Thus eightfold cross-validation was used for this study.

**Figure 2:**
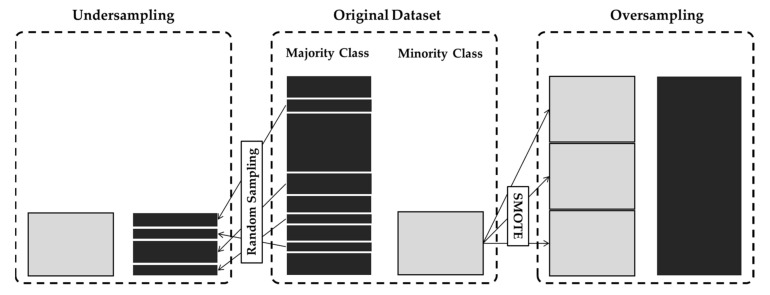
Undersampling and oversampling in the training set. In undersampling, the data belonging to the majority class were randomly partitioned to have the same length as the minority class. In oversampling, the data in the minority class were permuted and replicated to have the same length as those of the majority class.

In training, the imbalance of data between classes can adversely affect the ML training process. In this dataset, the average amount of data for driving was more than twice that for walking on level ground and 20 times that for descending or ascending stairs (Figure 1). To avoid the class imbalance problem, two training methods were adopted to balance the data: undersampling and oversampling (Figure 2). In undersampling, random epochs from the majority class were deleted without making changes to the data in the minority class. Random selection of the deleted epochs was repeated 10 times, and the performance was averaged for evaluation. In oversampling, randomly selected epochs in the minority class were replicated to balance the majority class. To reduce the overfitting problem, synthetic minority oversampling techniques (SMOTE), which create novel data based on the k-nearest neighbors of the minority class, were utilized instead of simple duplication [48]. These undersampling and oversampling procedures were only applied to the training and not the test dataset to avoid bias in ML.

### 2.2. Feature Extraction for the Machine Learning Approach

**Figure 3:**
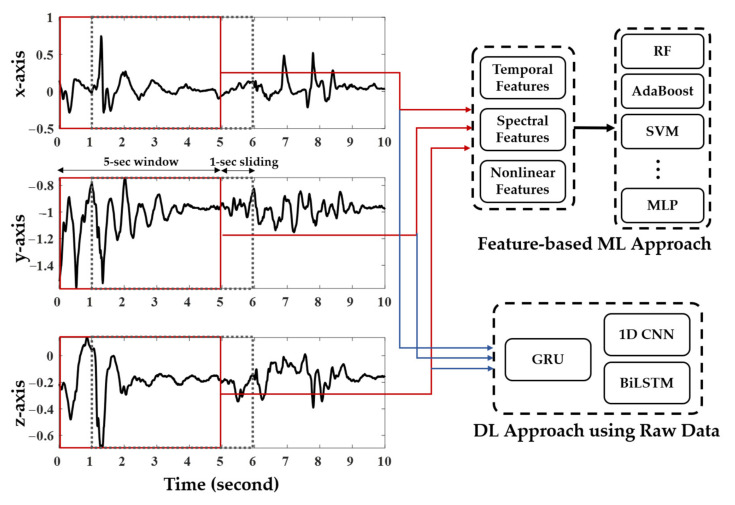
A 5-s window with a 4-s overlap was used to extract temporal, spectral, and nonlinear features, respectively. These features were used to construct 10 machine learning methods, including the ensemble approach. Four deep learning methods utilized raw data itself without feature extraction.

To identify the relevant features from the raw accelerometer data, three types of feature domains, which are frequently used for signal processing, were measured in this study: the temporal, spectral, and nonlinear domain features. All features were extracted from a 5-s window with a 4-s overlap so that the PA was classified every second (Figure 3). To reduce the subject variability of each feature in a cross-patient paradigm, all extracted features were normalized to have zero means and one standard deviation based on z-score standardization. Eight ML methods, including (1) RF, (2) AdaBoost, (3) DT, (4) k-NN, (5) linear discriminant analysis (LDA), (6) quadratic discriminant analysis (QDA), (7) support vector machine (SVM), and (8) MLP, were evaluated using the extracted features. In addition, to compare the influence of the feature extraction method, four types of DL methods including (1) gated recurrent unit (GRU), (2) bidirectional long short-term memory (BiLSTM), and (3) a one-dimensional CNN (1D CNN) were utilized to train the raw data.

#### 2.2.1. Temporal Features

In the temporal domain, eight features corresponding to the magnitude and statistical measures were calculated from each axis of the raw accelerometer data. The peak-to-peak amplitude, which subtracts the minimum value in the epoch from the maximum value, was used to estimate the intensity of the x, y, and z axes [36,44]. The root mean square (RMS) was also used to evaluate the absolute intensity instead of the mean value itself. The second, third, and fourth moments that represent the statistics of the data were also calculated in each epoch [49,50]. The second moment refers to the variance, which measures the variability of data distribution. In this study, a standard deviation for each epoch was used as a feature. The third and fourth moments denote the skewness and kurtosis that represent the properties of the data distribution. Two Hjorth parameters, namely statistical time domain measures known for their low computational costs, were used to measure the degree of complexity in the accelerometer data [51]. Hjorth mobility represents the mean frequency of the power spectrum. The Hjorth complexity estimates the change in frequency while mobility is proportional to the standard deviation of the power spectrum. Furthermore, the number of zero-crossing, which counts the number of intercepts with zero value in each epoch, was used to estimate the fundamental frequency [36]. The details of each feature are described in Table 1.

#### 2.2.2. Spectral Features

A short-time Fourier transform (STFT) was adopted to extract the spectral domain features. In the STFT, time series data were converted into a time–frequency domain using a fast Fourier transform (FFT) algorithm with a 5-s window and 1-s sliding window. The Hamming window was applied to minimize the side lobe. Then, 12 spectral features were extracted using power spectral densities (PSDs) corresponding to the 0–15-Hz frequency band obtained from the STFT (Table 2) [36,44,49,50]. Since PSDs were obtained for each 5-s window, the frequency resolution was 0.2 Hz, and thus 75 PSDs were selected as candidates for the spectral features. First, the total energy was obtained by summing all PSDs and entropy, which represents the randomness of PSDs [49]. Then, three peak powers with the largest power and the three dominant frequencies that corresponded to those peak powers were selected among the 75 PSDs. The dominant frequencies were sorted in ascending order. Furthermore, since several studies have reported that the frequency of the resting state and walking behavior each lie within 0.5 Hz and 1.5–2.5 Hz, the dominant frequency smaller than 2.5 Hz and their corresponding peak powers were used for this study [44,50]. The energy and entropy of PSDs smaller than 2.5 Hz were also included in the spectral feature set.

#### 2.2.3. Nonlinear Features

Nonlinear features were utilized to examine the inherent nonlinearity embedded in the accelerometer signals. The time delay parameter τ and embedding dimension d were used to reconstruct the time series data in the phase space [52]. The time delay parameter that represents the time lag to reconstruct an attractor in the phase space can be estimated using auto mutual information (AMI). Since mutual information (MI) quantifies the amount of information shared between two variables, AMI measures the autocorrelation between the original time series $x_{i}$ and the delayed time series $x_{i+\tau }$. The following equation describes the AMI function:

(1) $$AMI\left(x_{i},x_{i+\tau }\right)=\sum_{x_{i},\;x_{i+\tau }}^{}p_{xx}\left(x,x+\tau \right)\times log_{2}\left(\frac{p_{xx}\left(x,\;x+\tau \right)}{p_{x}\left(x_{i}\right),p_{x}\left(x_{i}+\tau \right)}\right),$$

where $p_{x}\left(x_{i}\right)$ and $p_{x}\left(x_{i}+\tau \right)$ are the probability functions of the original signal $x_{i}$ and time-delayed signal $x_{i+\tau }$, respectively, while $p_{xx}\left(x,x+\tau \right)$ indicates the joint probability of two signals. Then, the optimal time delay τ can be determined with an index of the first local minimum of the AMI. The embedding dimension, which is defined as the minimal number of data points to reconstruct signals in the phase space, can be calculated with the false nearest neighbor (FNN). The FNN determines the minimum embedding dimension by observing the changes in the nearby neighbors as the embedding dimension is increased from *m* to *m* + 1. If two points with a time delay τ are separated in a certain dimension, then it means the attractors in the phase space are not preserved. As a result, the first time index at a point where the FNN rate dropped to 0 and did not decrease in the higher dimension was determined as the embedding dimension. The FNN utilizes the equations below:

(2) $${\left[\frac{R_{m+1}{}^{2}\left(i,r\right)-R_{m}{}^{2}\left(i,r\right)}{R_{m}{}^{2}\left(i,r\right)}\right]}^{1/2}=\frac{\left|x_{i+m\tau }-x^{\left(r\right)}{}_{i+m\tau }\right|}{R_{m}{}^{2}\left(i,r\right)}>R_{tol},$$

(3) $$R_{m}{}^{2}\left(i,r\right)=\sum_{k=0}^{m-1}{\left[x\left(i+k\tau \right)-x^{\left(r\right)}\left(i+k\tau \right)\right]}^{2},$$

where $R_{m}{}^{2}\left(i,r\right)$ is the Euclidean distance in the *m*-dimensional space between $x_{i}$ and the *r*th nearest neighbor $x_{i}{}^{\left(r\right)}$ and $R_{tol}$ is the predefined tolerance threshold. Then, the time delay and the embedding dimension were used to calculate the Lyapunov exponent and sample entropy. The Lyapunov exponent λ quantifies the stability of the signals by estimating the divergence rate of two trajectories that were initially close to each other as in the following equation [53]:

(4) $$\lambda =\underset{i\to \infty }{lim}\frac{1}{m}\sum_{i=1}^{m}ln\left|\frac{\Delta \left(x_{i}-x_{i+\tau }\right)}{\Delta \left(x_{1}-x_{i+\tau }\right)}\right|,$$

The largest Lyapunov exponent is commonly used to determine the predictability of signals, and its positive, negative, and zero values refer to chaotic, steady, and periodic signals, respectively. The sample entropy (SampEn), a variant of the approximate entropy (ApEn), quantifies the complexity of the time series data [54]. Unlike ApEn, which finds the repetitive patterns of signals, SampEn eliminates self-matches, which makes it independent of the data length [55]. SampEn can be defined as follows:

(5) $$SampEn=-log(\frac{\sum_{i-1}^{N-m}number\;of\;x_{i}\;such\;that\;d\left[x_{i}{}^{m+1},\;x_{j}{}^{m+1}\right]<R}{\sum_{i-1}^{N-m}number\;of\;x_{i}\;such\;that\;d\left[x_{i}{}^{m},\;x_{j}{}^{m}\right]<R}),$$

(6) $$d\left[x_{i}{}^{m},\;x_{j}{}^{m}\right]=max_{k=1,2,\ldots ,m}\left(\left|x_{i+k-1}-x_{j+k-1}\right|\right),$$

where the function $d\left[x_{i}{}^{m},\;x_{j}{}^{m}\right]$ denotes the Chebyshev distance between two points that are not equivalent (*i* ≠ *j*). A larger SampEn means that the signal is more unpredictable and irregular. Finally, the Hurst exponent *H* measures the long-term memory of time series data [56]. It estimates the self-similarity of signals by fitting the power law as follows:

(7) $$Cn^{H}≅\;E\left[\frac{1}{\sigma }PTP(\sum_{i=1}^{n}(x_{i}-\mu ))\right],$$

where E indicates the expected value and *C* is constant. The function PTP denotes the peak-to-peak function that subtracts the minimum value from the maximum value, as described in Table 1. The value *H* being in the range of 0.5–1 indicates that the signals are persistently auto-correlated, while an *H* value of 0.5 implies that the signals are brown noise, meaning that they are completely uncorrelated.

### 2.3. Ensemble Learning

**Figure 4:**
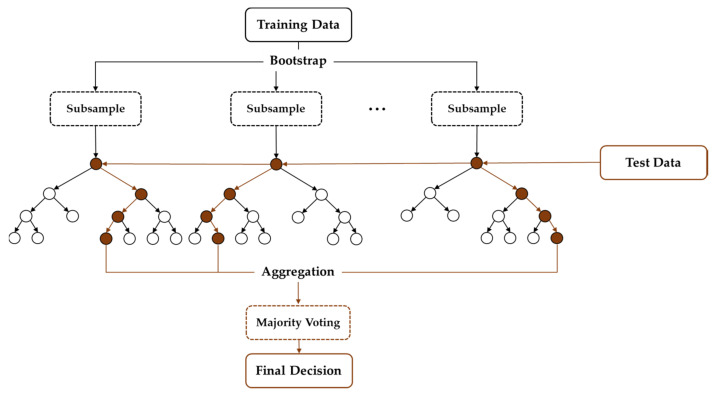
The structure of random forest (RF). RF is one of the bagging (bootstrap aggregating) methods that adopts bootstrapping and aggregation. In bootstrapping, RF generates multiple decision trees (DTs) using random subsets with the replacement of training data. In aggregation, RF labels the test data based on the class elected by majority voting from multiple trees.

Ensemble learning, which employs multiple models to make predictions on given data, aims to compensate for the potential problems that could arise from using a single classifier. Previous studies have shown that ensemble learning can mitigate the class imbalance problem which is common in machine learning, where classifiers can easily develop a bias toward the majority class [57]. The use of multiple learners in ensemble learning can also lower the risk of getting stuck in a local minimum, which is common when using an individual learner. The basic assumption is that the final output generated by ensemble learning leads to better prediction than that of the individual classifiers. Ensemble learning can be implemented in a sequential or parallel process. RF is one of the ensemble techniques, first introduced by Breiman in 2001, which uses bootstrap aggregation (bagging) to construct ensembles to deal with classification and regression problems [58]. It utilizes ensemble learning in a parallel process in which predictions made from multiple classifiers, called decision trees (DTs), are averaged to yield the final output (Figure 4). Each training data are randomly drawn from the original data set so that input variation is given to each learner. Each of the DTs starts with a root node, where a feature is used as a threshold to split the data into two branches, giving it a tree-like shape [59]. The same step is repeated using different features until the leaf node of the tree is reached, where a prediction of the class label is made. While the conventional DT is prone to overfitting the training data, RF is less likely to overfit due to its ensemble design. In RF, the data are randomly split into training and validation sets. For each set, decision trees are generated through bagging, which is a procedure of repeatedly drawing random samples from the dataset. Then, the final class label is determined by employing a majority vote, which means that the class label estimated by most of the trees is given as the final prediction. This majority vote system helps reduce the variance in predictions. A previous study has also shown that RF performed the best among a total of 11 classification algorithms, even with noisy and imbalanced data [60].

**Figure 5:**
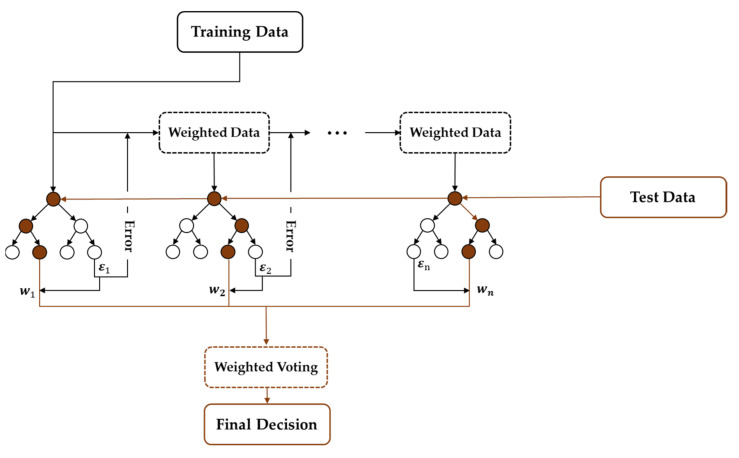
The structure of adaptive boosting (AdaBoost). AdaBoost generates multiple weak learners. Unlike RF, previous decision trees (DTs) affect the next DT by using the classification errors to weigh the misclassified data. The final decision is performed with weighted voting from multiple trees based on the error of each tree.

Boosting is another ensemble learning method, which sequentially generates multiple models based on the errors of the previous model (Figure 5). One of the most popular boosting algorithms is AdaBoost, where each decision tree in the ensemble is assigned a weighted error rate based on the previous model, which is used to determine the decision power of each tree [61]. Unlike RF, where the learners are independently trained with random subsamples, AdaBoost trains individual learners with the entire data in a serial manner by increasing the error weights of misclassified instances. Each learner is also assigned another set of learner weights which is inversely correlated with the assigned error weights. Thus, the larger the weighted error rate assigned to a tree, the less influence the tree has in majority voting for the final prediction. In this study, after testing for the optimal parameters, the number of decision trees was set to 100 for both RF and AdaBoost.

### 2.4. Evaluation Metrics

In this study, the influences of the undersampling and oversampling methods for balancing classes were examined based on the classification performance of ensemble learning, including RF and AdaBoost. Furthermore, the ensemble methods were compared to other ML models that are frequently exploited for various classification tasks. In most ML and DL studies, classification performance is assessed using the sensitivity, specificity, precision, F1-score, and accuracy. Sensitivity, which is also known as recall, measures the ratio between true positives (TP) and actual positives (TP + FN, where FN refers to false negatives). Therefore, it evaluates the performance of the model based on its ability to correctly recognize the target. Specificity measures the percentage of true negatives (TNs) out of the actual non-target cases (TN + FP), where FP refers to false positives), indicating the model’s ability to correctly classify non-target classes. Specificity is inversely proportional to sensitivity in that it increases as the sensitivity decreases and vice versa. Another evaluation metric is precision, which refers to the fraction of the actual target class (TP) among those classified as the target class (TP + FP). Next is the F1-score, which combines precision and recall by taking their harmonic mean, which is also frequently used for evaluating ML classifiers. Finally, there is the accuracy, the most commonly used evaluation metric, which calculates the ratio of correctly predicted cases (TP + TN) to the total predictions (TP + TN + FP + FN).

However, when evaluating the performance for imbalanced data, F1-score and accuracy can be unreliable, since they do not take the data distribution into account. Therefore, this study mainly adopted balanced accuracy, which can be defined as the average of the recall obtained from each class, for evaluating the multi-class ML classifiers. To compare the classification performance depending on the undersampling or oversampling approach or the classification performance among multiple ML classifiers, the Kruskal–Wallis test was utilized for each evaluation metric. Furthermore, multiple comparison problems were corrected using the Tukey–Kramer method.

## 3. Results

### 3.1. Classification Performance Depending on Undersampling or Oversampling

**Figure 6:**
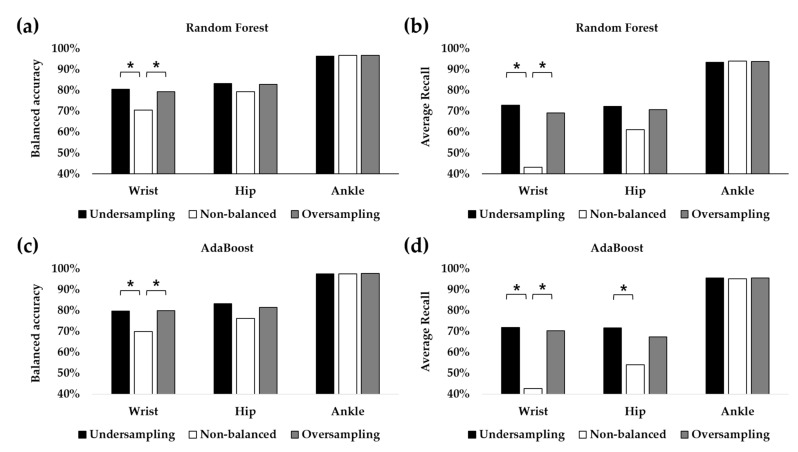
Classification performance of RF and AdaBoost depending on the undersampling and oversampling approach. (**a**) Balanced accuracies of RF. (**b**) Average recall of RF for two minority classes: ascending stairs and descending stairs. (**c**) Balanced accuracies of AdaBoost. (**d**) Average recall of AdaBoost for two minority classes. The asterisk (*) denotes *p* < 0.05 after post hoc analysis.

The balanced accuracies of RF and AdaBoost when using the original dataset with imbalanced classes were compared with those after applying the undersampling approach using random sampling and the oversampling approach using SMOTE methods (Figure 2). When using a wrist-worn sensor, the performances of both RF and AdaBoost in the classification of four PAs, including walking on level ground, descending stairs, ascending stairs, and driving, were highly affected (Figure 6). When using RF, the balanced accuracies in undersampling and oversampling were 80.45% and 79.36%, respectively, which were significantly larger than 70.55% with a non-balanced dataset (Figure 6a). When using AdaBoost, significant improvement after undersampling and oversampling was also observed (Figure 6c). Since deterioration of the classification performance with an imbalanced dataset was mainly observed in the minority classes, we examined the recalls (sensitivities) for ascending and descending stairs. The average recall of these two PAs in RF was 43.18% without the undersampling or oversampling methods. It significantly increased to 72.95% with the undersampling method and 69.14% with the SMOTE methods (Figure 6b). The average recalls were also significantly higher with undersampling (71.81%) and SMOTEs (70.34%) compared with that with unbalanced data (42.66%) (Figure 6d).

When using a hip-worn sensor, balanced accuracies were also enhanced with the undersampling and oversampling methods, but no significant difference was found (Figure 6a,c). The maximum accuracy was found in RF with undersampling, whose balanced accuracy was 80.45%. This result was approximately 10% larger than the accuracy with unbalanced data. The average recalls of the minority classes (i.e., ascending stairs and descending stairs) increased by approximately 30% when undersampling methods were applied (Figure 6b,d). In particular, the average recall with undersampling was significantly larger than that without balancing techniques when using AdaBoost (Figure 6d).

However, with an ankle-worn sensor whose classification performance between four activities was larger than 95% in most cases, the undersampling and oversampling methods did not affect classification performance significantly. Although the balanced accuracy in AdaBoost was the highest at 97.70% using SMOTE methods, it was not significantly larger than that with unbalanced data or with the undersampling method, which resulted in 97.47% and 97.48% accuracy, respectively (Figure 6b). The balanced accuracies of RF, whose best accuracy was 96.90%, also exhibited similar tendencies (Figure 6a).

**Figure 7:**
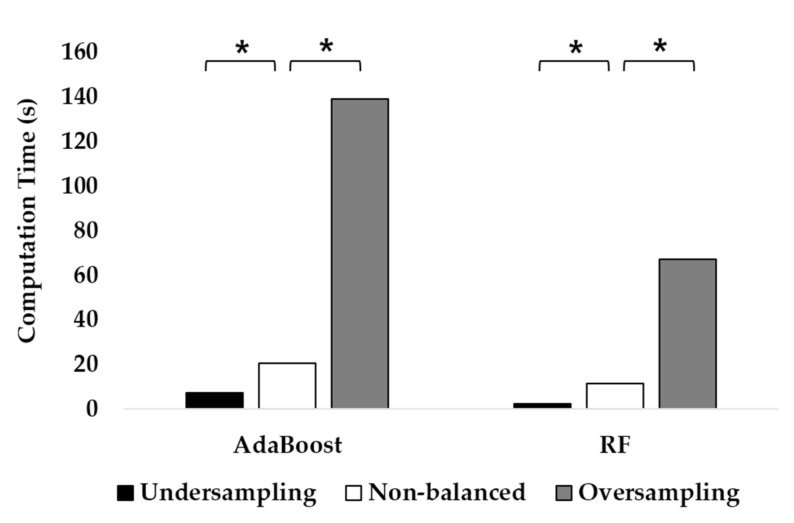
The computational cost of RF and AdaBoost when using the undersampling and oversampling approaches. The asterisk (*) denotes *p* < 0.05 after post hoc analysis.

To compare the computational costs of undersampling and oversampling, the time spent to train the two ensemble models was recorded for each eight times. The function *fitcensemble* was used to train RF and AdaBoost in MATLAB version 2021b. The number of learning cycles, which denotes the number of trees, was fixed at 100. The detailed specifications of the PC used for training are as follows: Microsoft Windows 10, AMD Ryzen 7 3800XT 8-core processor, 32 GB RAM, and an NVIDIA GeForce RTX 3060 Ti GPU. The number of epochs oversampled with the SMOTE method (138,310 on average) was approximately 22.3 times that of the random undersampling (6016.5 on average) and 2.8 times that of the non-balanced data (48,720 on average). When RF was utilized, the average computational cost of oversampling was 66.97 s, which was approximately 27.2 times that of undersampling, which was 2.46 s (Figure 7). As for AdaBoost, the ratio of computational costs between oversampling and undersampling was approximately 18.51. Furthermore, computational cost can be reduced in the undersampling approach compared with the originally unbalanced data. The computational cost of AdaBoost was approximately twice that of RF in the oversampling approach and three times that of RF in the undersampling approach.

### 3.2. Comparison of ML and DL Classifiers

Ensemble methods including RF and AdaBoost were compared to other ML models that are frequently exploited for various classification tasks. Six ML classifiers, including DT, k-NN, LDA, QDA, SVM, and MLP, utilized the same feature set used in RF and AdaBoost. For the k-NN algorithm, through optimization of the model, *k* was set to three. For the SVM, the linear kernel was selected instead of the Gaussian and the polynomial kernel through the optimization process. MLP only adopted a single layer because it utilizes the extracted feature set. On the other hand, three DL classifiers including GRU, BiLSTM, and a 1D CNN utilized the raw accelerometer data as input data. Through hyperparameter optimization, all three DL classifiers had two layers, while the number of filters and kernel size were 32 and 15 for the CNN, respectively, and the number of hidden units was 100 for GRU and BiLSTM. The maximum number of epochs was 100 for oversampling and 300 for undersampling.

**Figure 8:**
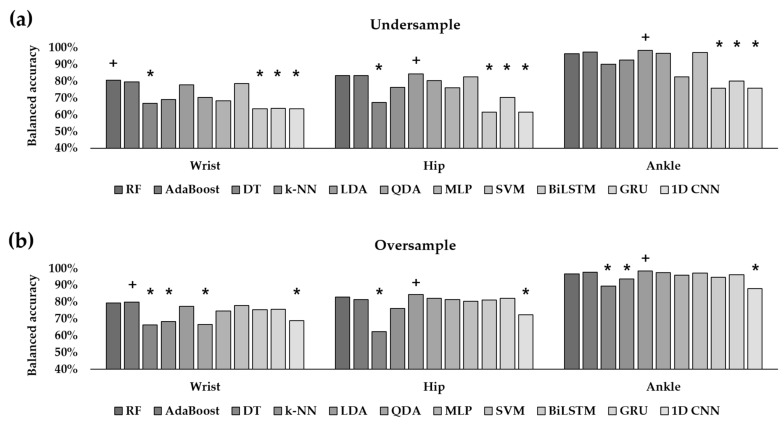
Balanced accuracies of eight feature-based ML classifiers and three DL classifiers in the (**a**) undersampling approach and (**b**) oversampling approach. The cross mark (+) denotes the classifier that exhibited the best performance. The asterisk (*) denotes the classifiers which were significantly different (*p* < 0.05) from the best classifier after post hoc analysis.

Most feature-based ML classifiers exhibited the best performance with the ankle accelerometer (Figure 8). When using an ankle-worn sensor, as described in Section 3.2., the balanced accuracies in all ML classifiers except DT and k-NN were larger than 90% with or without data balancing techniques. With the undersampling approach, the performance of the DL classifiers, including BiLSTM, GRU, and the 1D CNN, significantly decreased compared with that of the feature-based ML classifiers, including RF, AdaBoost, LDA, QDA, and SVM (Figure 8a). With the oversampling approach, the classification performance of BiLSTM and GRU was similar to that of LDA, RF, and AdaBoost (Figure 8b). Although the classification performance of the wrist and hip accelerometers was relatively lower than that of the ankle accelerometer, several ML classifiers achieved approximately 80% balanced accuracy. When a wrist sensor was utilized, RF resulted in the largest balanced accuracy of 80.45% with an undersampling approach (Figure 8a). Similar to the results of the ankle-worn sensor, those of the wrist-worn sensor showed that RF outperformed three DL classifiers with an undersampling approach. With the oversampling approach, the performance of DT, k-NN, QDA, and the 1D CNN was significantly lower than that of RF. When a hip-worn sensor was utilized, LDA exhibited the best balance accuracy of 84.43% with the oversampling approach. However, it was not significantly different from the results of other ML models such as RF (82.84%) and AdaBoost (81.43%). When the undersampling approach was adopted, LDA also exhibited the best accuracy of 84.27%, while RF and AdaBoost exhibited 83.34% and 83.28% accuracy, respectively.

### 3.3. Feature Importance Depending on Sensor Location

In this study, the PA classification was performed using three different sensor locations: the ankle, wrist, and hip. Furthermore, we extracted various types of features from the temporal, spectral, and nonlinear domains for the x, y, and z axes, which are widely used in PA classification and time series analysis. Because the four PAs included walking types and sedentary behaviors, which are highly associated with the ankle motion, classification performance was the best with the ankle-worn sensors. Meanwhile, the hip- and wrist-worn sensors exhibited relatively lower performance compared with the ankle-worn sensor because the mechanisms of the hip and wrist during walking activities were different from that of the ankle. To investigate the difference depending on sensor location and the types of features, we examined the features that highly affected the classification performance of each classifier. Since both RF and AdaBoost adopt DT, which uses multiple binary decision criteria (leaf nodes) to split data into two subsets (branches), the feature importance could be estimated with the criterion. The leaves and branches of each DT are grown by minimizing the Gini impurity. The Gini impurity of a node $N_{k}$ is defined as follows:

(8) $$Gini\left(N_{k}\right)=\sum_{i}^{}p_{i}\left(1-p_{i}\right)=1-\sum_{i}^{}p_{i}{}^{2},$$

where $p_{i}$ is the probability of the samples belonging to class *i*. If all samples belong to only one class, the Gini impurity in this node is zero; otherwise, it has a positive value. Then, the feature importance *I* can be obtained by examining the difference in the Gini impurity between the parent node and the two child nodes as follows:

(9) $$I\left(N_{k}\right)=\frac{\left(p_{k}\times Gini\left(N_{k}\right)-p_{kl}\times Gini\left(N_{kl}\right)-p_{kr}\times Gini\left(N_{kr}\right)\right)}{Total\;Number\;of\;Nodes},$$

where $p_{k}$, $p_{kl}$, and $p_{kr}$ refer to the probability at the parent node $N_{k}$ and two child nodes $N_{kl}$ and $N_{kr}$.

**Figure 9:**
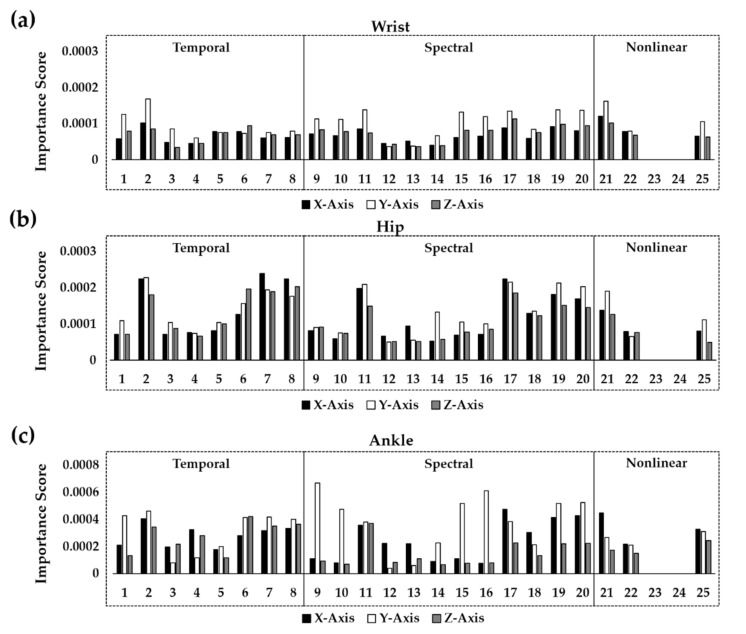
Feature importance of RF binary-class classification depending on the sensor location: (**a**) wrist-worn sensor, (**b**) hip-worn sensor, and (**c**) ankle-worn sensor. The indices in the horizontal line indicate (1) RMS, (2) σ, (3) skewness, (4) kurtosis, (5) the number of ZC, (6) PTP, (7) Hjorth mobility, (8) Hjorth complexity, (9–11) three PPs, (12–14) three DFs, (15) TE, (16) SpecEn, (17–20) PP, DF, TE, and SpecEn in the range of 0–2.5 Hz, (21) Hurst exponent, (22) AMI, (23) FNN, (24) Lyapunov exponent, and (25) SampEn.

In this study, the average importance level of each feature was evaluated from the RF model with an undersampling approach, which was accurate and fast. In general, the y axis of the accelerometer data played an important role when the wrist-worn or ankle-worn sensor was adopted (Figure 9). In the classification using the wrist accelerometer, the standard deviation (σ) and Hurst exponent of the y axis exhibited the largest importance scores (Figure 9a). The importance scores of the spectral features on the y axis such as the peak power (PP) and total energy (TE) were larger than those on other axes. In particular, the TE and spectral entropy (SpecEn) in the range of 0–2.5 Hz exhibited large scores on the y axis. In the classification using ankle accelerometer data, spectral features including the PPs, TE, and SpecEn along with the y axis showed the largest importance scores (Figure 9b). Similar to the results from the wrist-worn sensors, the TE and SpecEn in the range of 0–2.5 Hz along the y axis played important roles in PA classification. Unlike the wrist and ankle accelerometers, where the features along the y axis were significant, when the hip accelerometer was utilized, those along the x and z axes were also useful for classification (Figure 9c). Although the spectral features of the y axis, particularly in the range of 0–2.5 Hz, were still useful, the features of the x and z axis also exhibited similar importance scores. In particular, the Hjorth parameters of the x axis resulted in a large importance score.

## 4. Discussion

This study aimed to distinguish four PAs using raw accelerometer data collected from three different body parts: the wrist, hip, and ankle. Two ensemble learning methods of RF and AdaBoost were compared with other comparative ML and DL models in terms of differentiating sedentary behavior (driving) and three types of walking modalities (walking on level ground, ascending stairs, and descending stairs). Since the previous study on this dataset reported lower performance in the group-level classification compared with that in the subject-specific paradigm, this study aimed to improve the classification performance in the group-level paradigm [39]. Furthermore, to overcome the data imbalance problem in different PA types which deteriorates the classification performance, undersampling and oversampling methods were adopted in this study (Figure 2). Feature-based ML classifiers including RF and AdaBoost exhibited enhanced performance by solving the data imbalance problem using undersampling and oversampling methods (Figure 6). In the previous study that utilized the same dataset with DT, the sensitivity of the minority class was poor despite the high sensitivity of the majority class [39]. They reported that the sensitivities of ascending stairs and descending stairs in the group-level paradigm were approximately 40–55% when using wrist or hip sensors. The sensitivities of these minority classes ranged between 60% and 70% even with the data from the ankle-worn sensor. In this study, we demonstrated that the balanced accuracy and average recall of the minority classes were much higher than in the previous study. When using the ankle accelerometer, both the balanced accuracy and average recall were higher than 90%. When using the wrist or hip accelerometer, the balanced accuracy was approximately 80%, and the average recall of the minority classes was larger than 70%. Alharbi et al. also suggested that oversampling methods, including SMOTE and its variations, could enhance the classification of the minority class with different datasets [62]. For example, the classification accuracy of ascending stairs, which accounts for 6% of the PAMAP2 dataset, was 45.9% without oversampling but was enhanced to 60% with oversampling. However, they did not investigate the effect of undersampling on the classification performance.

The improvement in classification was not only due to the data balancing techniques but also the improved classifiers and the abundance of features. In Figure 8, among the feature-based ML classifiers, DT exhibited the lowest classification performance. While single DT is vulnerable to overfitting, ensemble methods such as RF and AdaBoost that utilize multiple trees are robust to noise and outliers. Although other ML models like LDA outperformed the ensemble methods with hip-worn sensors, the ensemble methods exhibited moderate performance regardless of the sensor locations while examining the influence of each feature on the classification criteria. Unlike the previous study on the same dataset, which extracted features from the vector magnitude that combined the three axes, this study extracted 25 temporal, spectral, and nonlinear features, which were used in PA classification as well as biosignal analysis along each of the three axes. Therefore, we utilized 75 features to train each classifier. By examining the feature importance collected from RF, we found that the features along the y axis were useful in classifying the walking types and sedentary behaviors when using the ankle- or wrist-worn sensors. In particular, the standard deviation and spectral features in the range of 0–2.5 Hz played important roles. Unlike these two sensors, where the features along the y axis were significant, when using the hip-worn sensor, the features along all three axes played important roles. This could be due to the more complex movement of a hip joint compared with that of a wrist or ankle during walking activities.

The features extracted from the ankle accelerometer data resulted in a higher classification performance than those from the hip- and wrist-worn sensors (Figure 6). Therefore, rather than hip- or wrist-worn sensors, ankle accelerometers which can be embedded in shoes are more adequate for differentiating different walking activities. Nonetheless, PA classification with hip- and wrist-worn sensors can also be conveniently measured, since the former can be embedded in a smartphone which can be placed in a pants pocket, and the latter can be embedded in a smartwatch. Since most PAs require arm and foot movement, the combination of multiple wearable sensors will be useful for differentiating diverse activities. For example, a study that utilized the WISDM dataset successfully classified 18 PAs, including walking types and household activities [34].

Although many studies reported that DL approaches were useful for the multi-class classification of PAs, the durations of each PA were balanced in most datasets. Since DL models require a substantial amount of data for each class, it is difficult to apply them in a free-living situation, where data imbalance between PAs is common. In a free-living situation, a larger portion of most people’s time is spent in sedentary behaviors like driving or walking on level ground instead of ascending or descending stairs. In this study, the performance of the DL models severely deteriorated with the undersampling approach. On the other hand, when using ML classifiers, the classification performance did not differ between the undersampling and oversampling methods. Since the computational cost was dramatically reduced with the undersampling method, ML classifiers including RF and AdaBoost, which were robust for the undersampled data, were more efficient if relevant features could be extracted (Figure 7). With the oversampled data, the performance of the DL models was enhanced to a level comparable to some ML models (Figure 6). If sufficient data were collected from each PA, the DL models would have demonstrated great performance in various classification tasks. For example, Ronald et al. achieved 95.09% accuracy in the classification of six activities using the Inception-ResNet-based deep learning model (iSPLInception) even in the between-subject paradigm [63]. Despite the great performance, DL models require a high computational cost. In this study, the average training time of the GRU model which only used two hidden layers was approximately 74 times that of RF.

In future studies, the classification performance of ensemble learning on the accelerometer data from more diverse PAs can be compared with that of other ML and DL models. Since this study classified only four PAs highly related to lower limb motion, the classification accuracy was almost perfect using the ankle-worn sensors. Meanwhile, the performance declined when using hip- or wrist-worn sensors. Therefore, to obtain good performance in the classification of more diverse PAs, the development or adoption of novel features to capture the distinct patterns of each PA is necessary. To overcome the limitation of this study, the DL approach, which can automatically capture distinct patterns of the data, can be applied in future studies. Since the DL approaches in this study only utilized two layers, a more advanced DL model with varying hyperparameters must be tested for potential improvement in performance. Finally, the DL models can be improved with sufficient accelerometer data for each PA as well as more advanced data augmentation techniques, such as a generative adversarial network (GAN).

## 5. Conclusions

This study proposes that ensemble learning methods are more robust in small datasets and subject variability compared with other various ML and DL models. In particular, the use of an RF classifier with an undersampling approach which has a low computational cost exhibited high classification performance even with the reduced data size. This can be useful in PA classification, since data imbalance problems between classes can easily appear in the free-living situation. Automatic PA classification will contribute to the enhancement of users’ health by providing a more precise estimation of energy expenditure and appropriate feedback on their PA status based on the classification outcome.

## Figures and Tables

**Table 1:** Temporal features extracted from the x, y, and z axes of the accelerometer data. The variables $x_{i},\;n,\;$ and μ each refer to the accelerometer data along the temporal axis, the total number of epoched data, and the average of each epoch, respectively. The symbol Δ denotes the first derivative, and the function *sgn* indicates the signum function that returns 0 if the input is equal to 0, 1 if the input is positive, and −1 if the input is negative.

Feature	Equation
Peak-to-Peak (PTP)	$PTP=max\left(x_{i}\right)-min\left(x_{i}\right)$
Root Mean Square (RMS)	$RMS=\sqrt{\frac{1}{n}\overset{n}{\underset{i=1}{\sum }}x_{i}{}^{2}}$
Standard Deviation (σ)	$\sigma =\sqrt{\frac{1}{n}\overset{n}{\underset{i=1}{\sum }}{\left(x_{i}-\mu \right)}^{2}}$
Skewness	$skewness=\frac{1}{n\times \sigma^{3}}\overset{n}{\underset{i=1}{\sum }}{\left(x_{i}-\mu \right)}^{3}$
Kurtosis	$kurtosis=\frac{1}{n\times \sigma^{4}}\overset{n}{\underset{i=1}{\sum }}{\left(x_{i}-\mu \right)}^{4}$
Hjorth Mobility	$Mobility=\frac{1}{\sigma }\sqrt{\frac{1}{n}\overset{n}{\underset{i=1}{\sum }}{\left(\Delta x_{i}-\mu_{d}\right)}^{2}}$
Hjorth Complexity	$Complexity=\frac{Mobility\left(\Delta x_{i}\right)}{Mobility\left(x_{i}\right)}$
Zero-Crossing (ZC)	$ZC=\frac{1}{2}\overset{n}{\underset{i=1}{\sum }}\left|sgn\left(x_{i}\right)-sgn\left(x_{i-1}\right)\right|$

**Table 2:** Spectral features extracted from the x, y, and z axes of accelerometer data. Each feature was extracted from two sets of the PSDs, $X_{f}$ was obtained using STFT: one in the frequency range of 0–15 Hz and another in the frequency range of 0–1.5 Hz. The function *argmax* indicates the index of the maximum value in the epoch.

Name	Equation	Number of Features	
		<15 Hz	<2.5 Hz
Total Energy (TE)	$Energy=\overset{k}{\underset{f=1}{\sum }}{\left|X_{f}\right|}^{2}$	1	1
Spectral Entropy (SpecEn)	$SpecEn=-\overset{k}{\underset{f=1}{\sum }}{\left|X_{f}\right|}^{2}log_{2}{\left|X_{f}\right|}^{2}$	1	1
Peak Power (PP)	$PP=max\left({\left|X_{f}\right|}^{2}\right)$	3	1
Dominant Frequency (DF)	$DF=argmax\left({\left|X_{f}\right|}^{2}\right)$	3	1

## Data Availability

The open database presented in PhysioNet was adopted for this study. The dataset can be found at https://doi.org/10.13026/51h0-a262 (accessed on 11 February 2022).
